# Supplementary material for: Phylogenetic and Evolutionary Studies of Grapevine Pinot Gris Virus Isolates from Canada
Source: Viruses. 2023 Mar 12;15(3):735. doi: 10.3390/v15030735 (PMC10057519; doi:10.3390/v15030735)
Supplement: Supplementary file 1 [file viruses-15-00735-s001.zip › viruses-2256927-supplementary.pdf]

Supplementary data

Manuscript Title: Phylogenetic and Evolutionary Studies of Grapevine Pinot Gris Virus Isolates from Canada

GPGV isolate	Accession no.	Symptoms	Cultivar	Location	Reference
S149	MK514532	Asymptomatic	Chardonnay	USA	[28]
S148	MK514531	Symptomatic	Sauvignon blanc	USA	
S145	MK514529	Asymptomatic	Cabernet sauvignon	USA	
S142	MK514528	Asymptomatic	N/A	USA	
S155	MK514527	Asymptomatic	N/A	USA	
S111	MK514526	Symptomatic	N/A	USA	
S107	MK514525	Asymptomatic	Cabernet sauvignon	USA	
S123	MK514524	Asymptomatic	Cabernet sauvignon	USA	
S106	MK514523	Symptomatic	Chardonnay	USA	
S105	MK514522	Symptomatic	Chardonnay	USA	
S104	MK514521	Asymptomatic	Chardonnay	USA	
S103	MK514520	Asymptomatic	Chardonnay	USA	
S147	MK514530	Asymptomatic	Zinfandel	USA	
S150	MK514533	Asymptomatic	Sauvignon Musque	USA	
S335	MK514534	Symptomatic	N/A	USA	
fvg-ls1	MH087439	Asymptomatic	Pinot blanc	Italy	[33]
fvg-ls6	MH087440	Asymptomatic	Pinot gris	Italy	
fvg-ls7	MH087441	Symptomatic	Pinot gris	Italy	
fvg-ls8	MH087442	Asymptomatic	Pinot gris	Italy	
fvg-ls12	MH087443	Symptomatic	Tocai fruilano	Italy	
fvg-ls13	MH087444	Asymptomatic	Tocai fruilano	Italy	
fvg-ls14	MH087445	Symptomatic	Pinot gris	Italy	
fvg-ls15	MH087446	Asymptomatic	Pinot gris	Italy	
fvg-ls17	MH087447	Asymptomatic	Glera	Italy	
SK30-1	KF686810	Asymptomatic	Vetliner	Slovakia	[23]
SK13	KF131425	Asymptomatic	Unknown	Slovakia	
SK01	KF131424	Asymptomatic	Unknown	Slovakia	
SK30	KF131423	Asymptomatic	Vetliner	Slovakia	
PN	KY706085	Symptomatic	Pinot gris	France	[14]
Mer	KM491305	Symptomatic	Merlot	France	
12G1110	MH802023	Symptomatic	N/A	Canada	N/A
SL13	KY747493	Asymptomatic	Bai-Ji-Xin	Pakistan	[21]
GR	MT414711	Symptomatic	Makripodia	Greece	N/A
TN	KT894101	Asymptomatic	Touriga Nacional	USA	[34]
IT	FR877530	Symptomatic	Pinot gris	Italy	[2]

Riesling	KX522755	Symptomatic	Riesling	Germany	[15]
FEM01	KU312039	Symptomatic	Silene latifolia	Italy	[46]
BC-1	KU194413	Symptomatic	Pinot gris	BC, Canada	[11]

Table S1: Accession numbers, symptoms, cultivar, origin and reference of 38 GPGV isolates used for genome analysis and gene-specific analyses obtained from NCBI,

Name on graph	Accession number	Symptoms	Cultivar	Location	Reference
ASG17 Italy	KU845342	Symptomatic	Glera	Italy	
ASG18 Italy	KU845343	Asymptomatic	Glera	Italy	[36]
ASG19 Italy	KU845344	Symptomatic	Glera	Italy	
ASG20 Italy	KU845345	Asymptomatic	Glera	Italy	
ASG21 Italy	KU845346	Symptomatic	Glera	Italy	
ASG22 Italy	KU845347	Asymptomatic	Glera	Italy	
ASG23 Italy	KU845348	Symptomatic	Glera	Italy	
ASG24 Italy	KU845349	Asymptomatic	Glera	Italy	
ASG25 Italy	KU845350	Symptomatic	Glera	Italy	
ASG26 Italy	KU845351	Asymptomatic	Glera	Italy	
ASG27 Italy	KU845352	Symptomatic	Glera	Italy	
ASG28 Italy	KU845353	Asymptomatic	Glera	Italy	
ASG29 Italy	KU845354	Symptomatic	Glera	Italy	
ASG30 Italy	KU845355	Asymptomatic	Glera	Italy	
ASG31 Italy	KU845356	Symptomatic	Glera	Italy	
ASG32 Italy	KU845357	Asymptomatic	Glera	Italy	
ASG33 Italy	KU845358	Symptomatic	Glera	Italy	
ASG34 Italy	KU845359	Asymptomatic	Glera	Italy	
ASG35 Italy	KU845360	Symptomatic	Glera	Italy	
ASG36 Italy	KU845361	Asymptomatic	Glera	Italy	
ASG39 Italy	KU845362	Symptomatic	Glera	Italy	
ASG40 Italy	KU845363	Asymptomatic	Glera	Italy	
ASG41 Italy	KU845364	Symptomatic	Glera	Italy	
ASG42 Italy	KU845365	Asymptomatic	Glera	Italy	
ASG43 Italy	KU845374	Asymptomatic	Glera	Italy	
ASG44 Italy	KU845376	Asymptomatic	Glera	Italy	
ASG45 Italy	KU845375	Asymptomatic	Glera	Italy	
ASG46 Italy	KU845377	Asymptomatic	Glera	Italy	
ASG47 Italy	KU845373	Symptomatic	Glera	Italy	
ASG48 Italy	KU845372	Symptomatic	Glera	Italy	
ASG49 Italy	KU845371	Symptomatic	Glera	Italy	
ASG50 Italy	KU845370	Symptomatic	Glera	Italy	
ASG51 Italy	KU845366	Symptomatic	Glera	Italy	
ASG52 Italy	KU845369	Symptomatic	Glera	Italy	
ASG53 Italy	KU845368	Symptomatic	Glera	Italy	
ASG54 Italy	KU845367	Symptomatic	Glera	Italy	
RQ25 Spain	MH019206	Symptomatic	Bobal	Spain	[26]
RQ30 Spain	MH019204	Symptomatic	Bobal	Spain	
RQ99 Spain	MH019205	Symptomatic	Macabeo	Spain	
RQ106 Spain	MH019203	Asymptomatic	Macabeo	Spain	
RQ108 Spain	MH019207	Symptomatic	Macabeo	Spain	
RQ111 Spain	MH019208	Symptomatic	Tintorena	Spain	
RQ113 Spain	MH019209	Asymptomatic	Tintorena	Spain	
RQ115 Spain	MH019210	Symptomatic	Tintorena	Spain	
RQ116 Spain	MH019211	Asymptomatic	Bobal	Spain	
RQ117 Spain	MH019212	Symptomatic	Bobal	Spain	
RQ125 Spain	MH019213	Asymptomatic	Tintorena	Spain	
RQ136 Spain	MH019214	Symptomatic	Bobal	Spain	

1 Czech	MW147697	Symptomatic	Sauvignon blanc / SO4	Czech Republic	[5]
2 Czech	MW147699	Symptomatic	Sauvignon blanc / SO4	Czech Republic	
4 Czech	MW147701	Symptomatic	Sauvignon blanc / SO4	Czech Republic	
6 Czech	MW147703	Symptomatic	Sauvignon blanc / SO4	Czech Republic	
8 Czech	MW147696	Symptomatic	Sauvignon blanc / SO4	Czech Republic	
MOLA10 Italy	LN606702	Asymptomatic	Supernova	Italy	[38]
MOLA6 Italy	LN606703	Asymptomatic	Supernova	Italy	
MOLA3x3 Italy	LN606704	Asymptomatic	Supernova	Italy	
MOLA14 Italy	LN606705	Asymptomatic	Supernova	Italy	
MOLA1 Italy	LN606706	Asymptomatic	Supernova	Italy	
3T Italy	LN606707	Symptomatic	Pinot gris	Italy	
FI8AV Italy	LN606708	Symptomatic	Traminer	Italy	
FI7AV Italy	LN606709	Symptomatic	Traminer	Italy	
FI6AV Italy	LN606710	Symptomatic	Traminer	Italy	
FI4N Italy	LN606711	Asymptomatic	Traminer	Italy	
FI4A Italy	LN606712	Symptomatic	Traminer	Italy	
FI3A Italy	LN606713	Symptomatic	Traminer	Italy	
BE5A Italy	LN606714	Symptomatic	Traminer	Italy	
BA-FA-P4 Italy	LN606715	Symptomatic	Traminer	Italy	
LBE-FA-P2 Italy	LN606716	Symptomatic	Traminer	Italy	
BE-ac- Italy	LN606717	Symptomatic	Traminer	Italy	
ZA505-9A Italy	LN606719	Symptomatic	Pinot gris	Italy	
NAV9181N Italy	LN606718	Asymptomatic	Pinot gris	Italy	
ZA505-5A Italy	LN606720	Symptomatic	Pinot gris	Italy	
Italy	LN606721	Symptomatic	Pinot gris	Italy	
ZA505-3N Italy	LN606722	Symptomatic	Pinot gris	Italy	
BE-FA59-1A Italy	LN606723	Symptomatic	Pinot gris	Italy	
ZA505-2N	LN606724	Asymptomatic	Pinot gris	Italy	
A505-2A	LN606725	Asymptomatic	Pinot gris	Italy	
ZA505-1N Italy	LN606726	Asymptomatic	Pinot gris	Italy	
ZA-PA-P4 Italy	LN606727	Symptomatic	Pinot gris	Italy	
ZA-PA-P1 Italy	LN606728	Symptomatic	Pinot gris	Italy	
ZA-PA-P2 Italy	LN606729	Asymptomatic	Pinot gris	Italy	
ZA505-10N Italy	LN606730	Symptomatic	Pinot gris	Italy	
ZA505-9A 13 Italy	LN606731	Symptomatic	Pinot gris	Italy	

ZA505-8N Italy	LN606732	Asymptomatic	Pinot gris	Italy	
ZA505-7N Italy	LN606733	Asymptomatic	Pinot gris	Italy	
ZA505-7A Italy	LN606734	Symptomatic	Pinot gris	Italy	
ZA505-6N Italy	LN606735	Asymptomatic	Pinot gris	Italy	
ZA505-6A Italy	LN606736	Symptomatic	Pinot gris	Italy	
LA-GO-P5 Italy	LN606737	Symptomatic	Pinot gris	Italy	
MER-FA-1A Italy	LN606738	Symptomatic	Merlot	Italy	
ALA-P4 Italy	LN606739	Symptomatic	Pinot gris	Italy	
ZA505-9N Italy	LN606740	Asymptomatic	Pinot gris	Italy	
ZA6Nclorosi Italy	LN606742	Asymptomatic	Pinot gris	Italy	
ZA505-8A Italy	LN606741	Symptomatic	Pinot gris	Italy	
Clair Algeri	MT272732	Asymptomatic	Clairette	Algeria	N/A
F4P5.M44 Moldova	LT719093	Asymptomatic	Margaritar	Moldova	[20]
HUCSK8 Hungary	MK953676	Asymptomatic	Not found	Hungary	N/A
HUCSK9s Hungary	MK953677	Asymptomatic	Not found	Hungary	
CF-BR Brazil	KY829001	Asymptomatic	Cab Franc	Brazil	[9]
ME-BR Brazil	KY829002	Asymptomatic	Merlot	Brazil	
SK53 Slovakia	KF134127	Asymptomatic	Muller Thurgau	Slovakia	[23]
KF134126 SK107 Slovakia	KF134126	Asymptomatic	Alibernet	Slovakia	
SK56 Slovakia	KF134129	Asymptomatic	Vetliner	Slovakia	
SK312 Slovakia	KF134128	Asymptomatic	Vetliner	Slovakia	
S149 USA	MK514532	Asymptomatic	Chardonnay	USA	[28]
S148 USA	MK514531	Symptomatic	Sauvignon blanc	USA	
S145 USA	MK514529	Asymptomatic	Cabernet sauvignon	USA	
S142 USA	MK514528	Asymptomatic	Unknown	USA	
S115 USA	MK514527	Asymptomatic	Unknown	USA	
S111 USA	MK514526	Symptomatic	Unknown	USA	
S107 USA	MK514525	Asymptomatic	Cabernet sauvignon	USA	
S123 USA	MK514524	Asymptomatic	Cabernet Sauvignon	USA	
S106 USA	MK514523	Symptomatic	Chardonnay	USA	
S105 USA	MK514522	Asymptomatic	Chardonnay	USA	
S104 USA	MK514521	Asymptomatic	Chardonnay	USA	
S103 USA	MK514520	Asymptomatic	Chardonnay	USA	
S147 USA	MK514530	Asymptomatic	Zinfandel	USA	

S150 USA	MK514533	Asymptomatic	S. Musque	USA	
S335 USA	MK514534	Symptomatic	Unknown	USA	
Gr Greece	MT414711	Symptomatic	Makripodia	Greece	N/A
PN France	KY706085	Symptomatic	Pinot gris	France	[14]
12G1110 Canada	MH802023	Asymptomatic	Unknown	Canada	[11]
SL13 Pakistan	KY747493	Asymptomatic	Bai Ji Xin	Pakistan	[21]
TN USA	KT894101	Asymptomatic	Touriga Nacional	USA	[34]
BC-1 Canada	KU194413	Symptomatic	Pinot gris	BC	[11]
fvg-Is1	MH087439	Asymptomatic	Pinot blanc	Italy	[33]
fvg-Is6 Italy	MH087440	Asymptomatic	Pinot gris	Italy	
fvg-Is7 Italy	MH087441	Asymptomatic	Pinot gris	Italy	
fvg-Is8 Italy	MH087442	Asymptomatic	Pinot gris	Italy	
fvg-Is12 Italy	MH087443	Symptomatic	Tocai Fruilano	Italy	
fvg-Is13 Italy	MH087444	Asymptomatic	Tocai Fruilano	Italy	
fvg-Is14 Italy	MH087445	Symptomatic	Pinot gris	Italy	
fvg-Is15	MH087446	Asymptomatic	Pinot gris	Italy	
fvg-Is17	MH087447	Asymptomatic	Glera	Italy	
IT Italy	FR877530	Symptomatic	Pinot gris	Italy	[2]
Riesling 25-3 Germany	KX522755	Symptomatic	Riesling	Germany	[15]
Mer France	KM491350	Symptomatic	Merlot	France	[14]
FEM01 Italy	KU312039	Symptomatic	Silene latifolia	Italy	[46]
SK30-1 Slovakia	KF686810	Asymptomatic	Vetliner	Slovakia	[23]
SK13 Slovakia	KF131425	Asymptomatic	Unknown	Slovakia	
SK01 Slovakia	KF131424	Asymptomatic	Unknown	Slovakia	
SK30 Slovakia	KF131423	Asymptomatic	Vetliner	Slovakia	
ON-1	OK558797	Asymptomatic	Chardonnay	Ontario	This study
ON-2	OK558798	Asymptomatic	Chardonnay	Ontario	
ON-3	OK558799	Asymptomatic	Chardonnay	Ontario	
ON-4	OK558800	Asymptomatic	Chardonnay	Ontario	
ON-5	OK558801	Asymptomatic	Chardonnay	Ontario	
ON-6	OK558802	Asymptomatic	Chardonnay	Ontario	
ON-7	OK558803	Asymptomatic	Chardonnay	Ontario	
ON-8	OK558804	Asymptomatic	Chardonnay	Ontario	
ON-9	OK558805	Asymptomatic	Chardonnay	Ontario	
ON-10	OK558806	Asymptomatic	Pinot noir	Ontario	
ON-11	OK558807	Asymptomatic	Pinot gris	Ontario	
ON-12	OK558808	Asymptomatic	Mourvèdre	Ontario	
ON-13	OK558809	Asymptomatic	Pinot gris	Ontario	

BC-2	OK558810	Symptomatic	Riesling	British Columbia
BC-3	OK558811	Asymptomatic	Merlot	British Columbia
BC-4	OK558812	Asymptomatic	Pinot gris	British Columbia
BC-5	OK558813	Asymptomatic	Cabernet franc	British Columbia
BC-6	OK558814	Asymptomatic	Cabernet franc	British Columbia
BC-7	OK558815	Asymptomatic	Cabernet franc	British Columbia
BC-8	OK558816	Asymptomatic	Cabernet franc	British Columbia
NS-1	OK558817	Asymptomatic	Vidal blanc	Nova Scotia
QC-1	QC-1	Asymptomatic	N/A	Quebec
QC-2	QC-2	Asymptomatic	N/A	Quebec
QC-3	QC-3	Asymptomatic	N/A	Quebec
QC-4	QC-4	Asymptomatic	N/A	Quebec
QC-5	QC-5	Asymptomatic	N/A	Quebec

Table S2: Accession numbers, symptoms, cultivar, origin and references of 169 GPGV isolates used in MP-CP overlapping region analysis obtained from NCBI

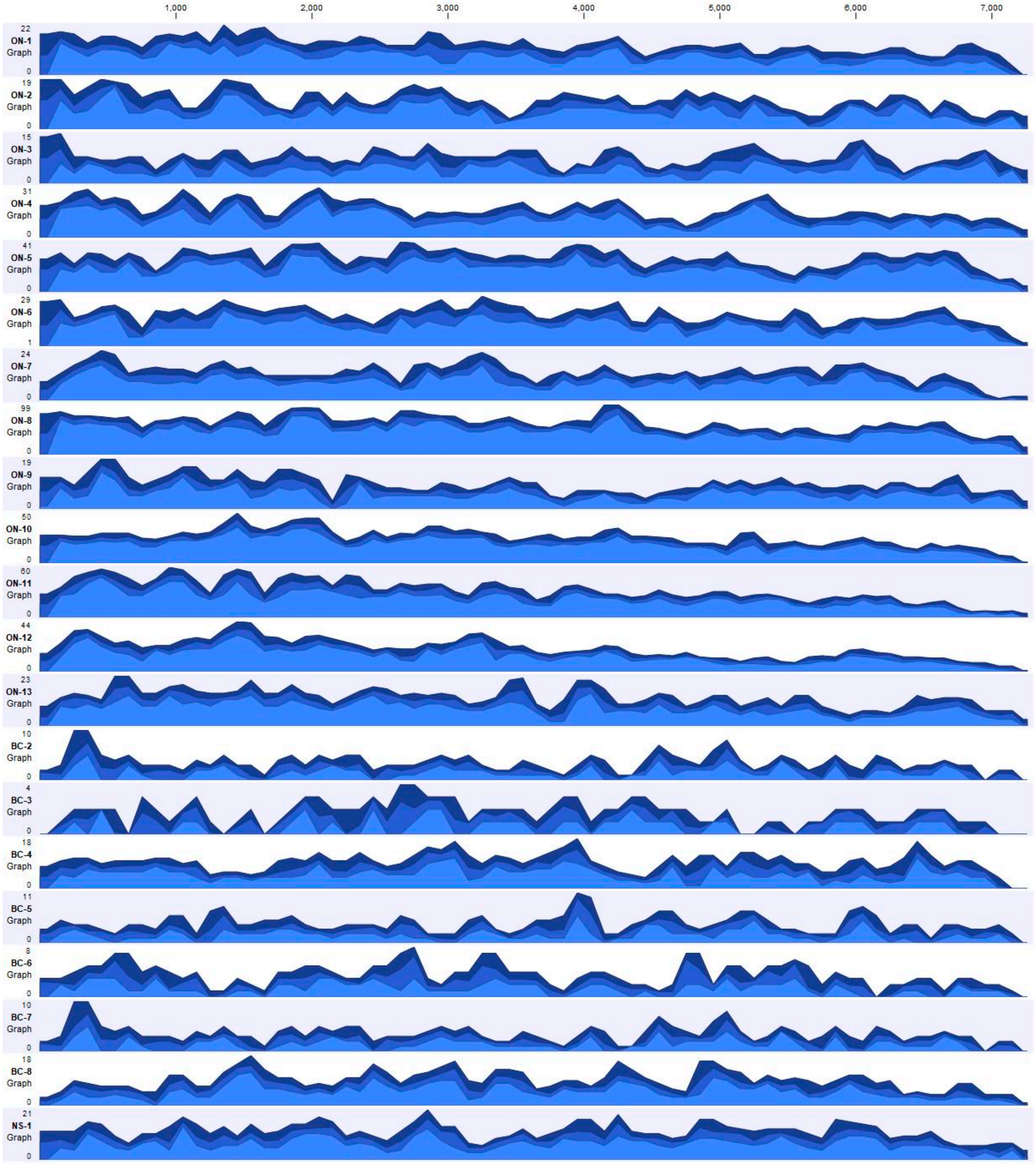

Figure S1: Depth and coverage of genome sequencing of Grapevine Pinot gris virus from 21 libraries comprised with CGW (CLC Genomics Workbench 20.0.4). Numbers on the top edge represent the length of the sequences while small numbers on the left edge represent depth. The blue area represents reads mapped on reference**.**

Table S3: Details on recombination events in Grapevine Pinot gris virus genome that were detected with seven methods using RDP4 software. Acronyms: R - Recombination Detection Program - RDP, G - GENECONV, B - Bootscan/Rescan, M - MaxChi, C - Chimaera, S - SisScan, T - 3Seq**.** Acronyms: R - Recombination Detection Program - RDP, G - GENECONV, B - Bootscan/Rescan, M - MaxChi, C - Chimaera, S - SisScan, T - 3Seq

**Event Number**	**Target**	**Major parent**	**Minor Parent**	**Detection methods**						
				**R[a]**	**G[b]**	**B[c]**	**M[d]**	**C[e]**	**S[f]**	**T[g]**
**1**	**fvg-Is8**	**Unknown**	**fvg-Is12**	+	+	+	+	+	+	-
**2**	**fvg-Is14**	**fvg-Is7**	**fvg-Is12**	+	+	+	+	+	+	+
**3**	**BC-5**	**BC-8**	**NS-1**	-	+	+	+	+	+	+
**4**	**fvg-Is17**	**FEM0**	**fvg-Is12**	+	+	-	+	+	+	+
5	fvg-Is7	PN	fvg-Is1	-	+	+	-	-	+	-
6	fvg-Is14	Unknown	fvg-Is7	-	+	+	+	-	+	-
7	BC-8	Unknown	NS-1	-	+	+	+	+	-	-
8	fvg-Is15	FEM0	ON10	-	+	-	-	-	-	-

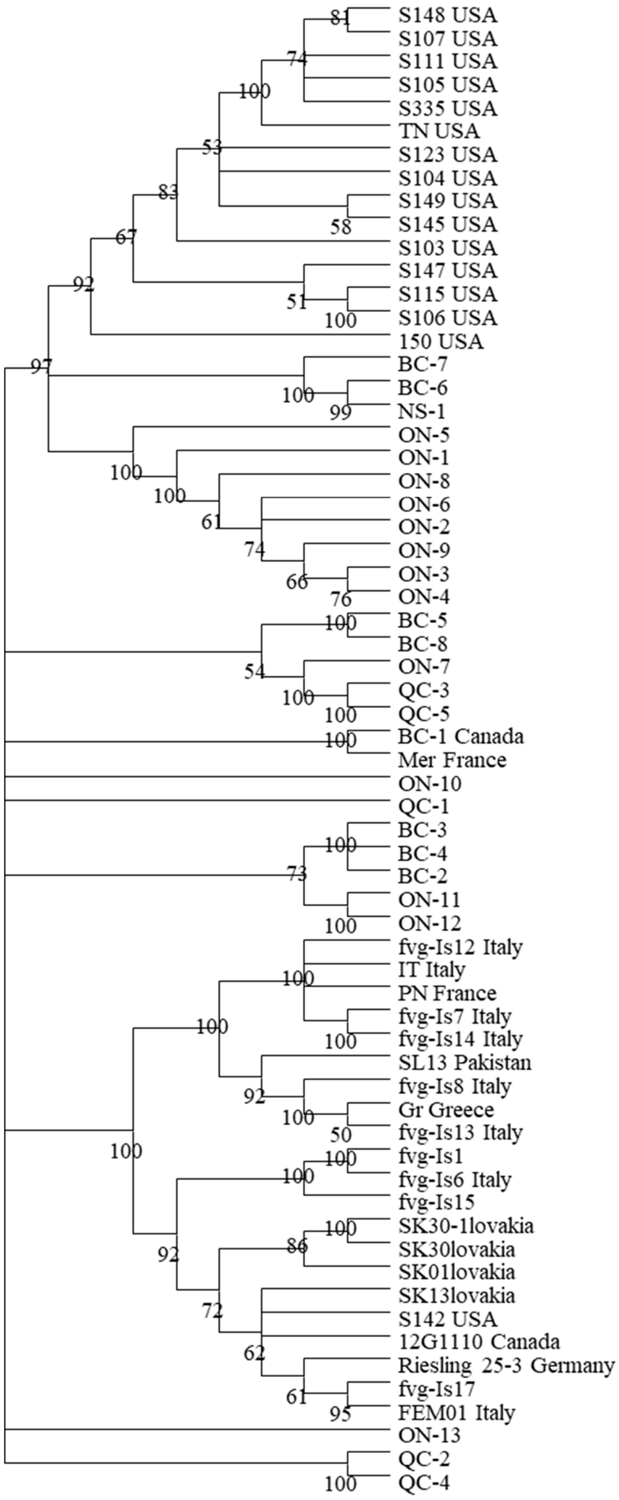

Figure S2: Unrooted maximum likelihood model for the largest recombination-free fragment for the 64 Grapevine Pinot gris virus isolates subjected in this study (4866bp, nt2293-nt7159 on

alignment). This tree was constructed with MEGA-X (General Time Reversal Model with discreet gamma distribution and invariant sites. Bootstrap value of 1000. Branches with less than 50% bootstrap consensus were collapsed. Details regarding this fragment and its breakpoints can be found in Table S3, S4 and S5.

Table S4: Details on recombination events in Grapevine Pinot gris virus genome that were detected with by seven methods using RDP4 software. The Even number column refers to the event number reported in Table S3 in the manuscript. Acronyms: R - Recombination Detection Program - RDP, G - GENECONV, B - Bootscan/Rescan, M - MaxChi, C - Chimaera, S - SisScan, T - 3Seq

Event number	Isolates involved	Beginning breakpoint	Ending breakpoint	p-values						
				R[a]	G[b]	B[c]	M[d]	C[e]	S[f]	T[g]
1	fvg-Is8 and fvg-Is12	2228	7172	1.38 E-7	4.09 E-10	2.90 E-14	5.59 E-8	2.06 E-8	3.02 E-12	5.75 E-21
2	Fvg-Is12 & Fvg-Is14	2292	7159	3.37 E-4	5.11 E-7	3.76 E-11	4.52 E-7	5.70 E-8	4.77 E-6	3.90 E-7
3	BC-5, BC-8, NS-1	2196	7192	7.53 E-5	5.22 E-6	1.78 E-10	8.25 E-11	8.55 E-3	4.31 E-13	7.73 E-15
4	Fvg-Is17 & Fvg-Is12	879	7171	2.24 E-4	1.11 E-7	4.20 E-11	6.66 E-7	5.70 E-8	2.77 E-6	6.91 E-7

Table S5: Recombination-free segments of genomes of grapevine Pinot gris virus isolates derived from predicted significant breakpoints. Details regarding the origin of these breakpoints can be found in table S4 above. Recombination-free genome fragments 200-bp sized or longer were reported in bold.

Segment	Beginning breakpoint	Ending breakpoint	Length (bp)
**1**	**0**	**896**	**896**
**2**	**897**	**2195**	**1298**
3	2196	2228	32
4	2229	2292	63
**5**	**2293**	**7159**	**4866**
6	7160	7162	2
7	7163	7172	9
8	7173	7289	116

Table S6: Estimates of evolutionary divergence over sequence pairs between clade for full genome of grapevine Pinot gris virus isolates using maximum likelihood model.

	Mean distance			
**Clade**	**1**	**2**	**3**	**4**
**1**	N/A	0.0010	0.0017	0.0015
**2**	2.23%	N/A	0.0015	0.0013
**3**	3.07%	3.04%	N/A	0.0015
**4**	3.02%	2.93%	2.80%	N/A

The percentage number below the diagonal line represent the percentage of base substitutions per site from averaging over all sequence pairs between group.. Standard error estimate(s) are shown above the diagonal and were obtained by a bootstrap procedure (1000 replicates). Analyses were conducted using the Maximum Composite Likelihood model [1]. This analysis involved 63 nucleotide sequences. No preference on start codon position was selected. All ambiguous positions were removed for each sequence pair (pairwise deletion option). There were a total of 7289 positions in the final dataset. Evolutionary analyses were conducted in MEGA X [2]

Table S7: Estimates of evolutionary divergence over sequence pairs within clade for the full genome of grapevine Pinot gris virus isolates using maximum likelihood model.

**Clade**	**Mean distance**	**Standard error**
**1**	1.37%	0.0007
**2**	2.08%	0.0008
**3**	1.93%	0.0016
**4**	2.06%	0.0012

The number of base substitutions per site from averaging over all sequence pairs within each group are shown. Standard error estimate(s) are shown in the second column and were obtained by a bootstrap procedure (1000 replicates). Analyses were conducted using the Maximum Composite Likelihood model [1]. This analysis involved 58 nucleotide sequences. No specific codon position was selected. All ambiguous positions were removed for each sequence pair (pairwise deletion option). There were a total of 7289 positions in the final dataset. Evolutionary analyses were conducted in MEGA X [2]

Table S8: Estimates of evolutionary divergence over sequence pairs between clades for the largest recombination-free fragment of grapevine Pinot gris virus using maximum likelihood model.

	Mean distance			
**Clade**	**1**	**2**	**3**	**4**
**1**	N/A	0.0012	0.0017	0.0018
**2**	2.02%	N/A	0.0017	0.0018
**3**	2.87%	2.85%	N/A	0.0017
**4**	3.05%	2.98%	3.01%	N/A

The percentage number below the diagonal line represent the percentage of base substitutions per site from averaging over all sequence pairs between group.. Standard error estimate(s) are shown above the diagonal and were obtained by a bootstrap procedure (1000 replicates). Analyses were conducted using the Maximum Composite Likelihood model [1]. This analysis involved 64 nucleotide sequences. No preference on start codon position was selected. All ambiguous positions were removed for each sequence pair (pairwise deletion option). There were a total of 4868 positions in the final dataset. Evolutionary analyses were conducted in MEGA X [2]

Table S9: Estimates of evolutionary divergence over sequence pairs within clade for the largest recombination-free grapevine Pinot gris virus fragment using the maximum likelihood model.

Clade	Mean distance	Standard error
**1**	1.31%	0.0007
**2**	1.80%	0.0010
**3**	1.88%	0.0017
**4**	2.08%	0.0015

The number of base substitutions per site from averaging over all sequence pairs within each group are shown. Standard error estimate(s) are shown in the second column and were obtained by a bootstrap procedure (1000 replicates). Analyses were conducted using the Maximum Composite Likelihood model [1]. This analysis involved 64 nucleotide sequences. No specific codon position was selected. All ambiguous positions were removed for each sequence pair (pairwise deletion option). There were a total of 4868 positions in the final dataset. Evolutionary analyses were conducted in MEGA X [2]

Table S10: Estimates of evolutionary divergence over sequence pairs between clade for MP-CP region of grapevine Pinot gris virus using maximum likelihood model.

Clade	1	2
**1**	N/A	0.0067
**2**	4.4%	N/A

The number of base substitutions per site from averaging over all sequence pairs between groups are shown. Standard error estimate(s) are shown above the diagonal and were obtained by a bootstrap procedure (1000 replicates). Analyses were conducted using the Maximum Composite Likelihood model [1]. This analysis involved 168 nucleotide sequences. No preference on start codon position was selected. All ambiguous positions were removed for each sequence pair (pairwise deletion option). There were a total of 549 positions in the final dataset. Evolutionary analyses were conducted in MEGA X [2]

Table S11: Estimates of evolutionary divergence over sequence pairs within clade for MP-CP regions of grapevine Pinot gris virus using maximum likelihood model.

Clade	Mean distance	Standard error
**1**	2.30%	0.0029
**2**	3.10%	0.0041

The number of base substitutions per site from averaging over all sequence pairs within each group are shown. Standard error estimate(s) are shown in the second column and were obtained by a bootstrap procedure (1000 replicates). Analyses were conducted using the Maximum Composite Likelihood model [1]. This analysis involved 168 nucleotide sequences. No preference on start codon position was selected. All ambiguous positions were removed for each sequence pair (pairwise deletion option). There were a total of 549 positions in the final dataset. Evolutionary analyses were conducted in MEGA X [2]
